# Pudendal Hematocele

**Published:** 1895-07

**Authors:** W. L. Patten

**Affiliations:** Milltown, Ga.


### PUDENDAL HEMATOCELE.

MILLTOWN, GA., May 13, 1895.

*To the Atlanta Medical and Surgical Journal:*

March 22, 1893, Mrs. M., age thirty-eight years, married, mother of seven children, was thrown from a buggy by a runaway horse. Wheel passed over left portion of pupendum, producing hematocele in region of left labium, with other but slight injuries over different portions of the body.

I reached her about three hours after the accident and made a diagnosis as stated above. It was by far the largest pudendal hematocele I have ever witnessed, fully as large as an ordinary fetal head.

At once I began firm pressure and cold applications, and ordered them kept up all night. As she was suffering great pain I administered a small dose of morphine hypodermatically, and left her for the night.

On my return next morning I found her resting well, having spent a very good night, with some reduction in size of tumor.

I continued this treatment for the next twenty-four hours, giving a saline purge in the meantime.

I then discontinued cold application and used an astringent lotion of lead, opium, and arnica. I continued this treatment for six days, noting a gradual decline in size of tumor and symptoms all good, no sign of suppuration or inflammation of the parts. As the symptoms were still good and swelling gradually getting less, I continued treatment four days longer, making ten days from reception of injury. The tumor was reduced to about half its original size. It now came to a standstill. The past treatment ceased to produce further improvement.

I thoroughly cleansed the parts, made a free incision, turned out a very large blood-clot, with more or less blood surrounding it, but no tendency to pus formation that I could tell. I thoroughly irrigated cavity with a warm antiseptic solution, packed it with iodoform gauze, and left the after-treatment for the family to carry out, instructing them to change dressing daily till it healed up.

I saw her no more for ten days, when I found her well and attending to her domestic affairs. She gave birth to twins a few weeks ago.

I am aware that in all cases we cannot wait so long as in this case before operating, but when the hematocele is so large, symptoms not urgent, no sign of pus formation, and even a very slight diminution in size of tumor under local and constitutional treatment, I think it advisable to defer operation as long as possible, for time certainly lessens the danger of an outbreak of hemorrhage of the original lacerated vessels that might cause trouble when the tumor is opened. W. L. PATTEN, M.D.

---

### DEATH RATE OF THE RACES.

In the reports of the eleventh vital statistics of the United States census the corresponding data from Boston, Philadelphia, Baltimore, Washington, and from the New England States as a whole, taken with those of New York State and New York city, and with those derived from a special investigatton of over 10,000 Jewish families, including over 50,000 persons, led to the following conclusions as being probable for the United States:

1. The colored race is shorter lived than the white and has a very high infantile death rate; it is specially liable to tuberculosis and pneumonia and less liable than the white race to malaria, yellow fever and cancer.

2. The Irish race has a rather low death rate among its young children, but a very high one among its adults, due to a considerable extent to the effects of tuberculosis, pneumonia, and alcoholism.

3. The Germans appear to be particularly liable to disorders of the digestive organs and to cancer.

4. The Hebrews have a low death rate and a more than average longevity; they are less affected than other races by consumption, pneumonia, and alcoholism, but are especially liable to diabetes, locomotor ataxia, and certain other diseases of the nervous system. —*Medical Record.*

